# A Multicenter Cross-sectional Study on the Prevalence and Impact Factors of Hysteria Tendency in the Eastern Chinese Adolescents

**Published:** 2018-12

**Authors:** Gang ZHAO, Li XIE, Yong XU, Qinglin CHENG

**Affiliations:** 1. Hangzhou Center for Disease Control and Prevention, Hangzhou, China; 2. Dept. of Adolescents and Children Health, School of Public Health, Medical College of Soochow University, Suzhou, China

**Keywords:** Hysteria tendency, Adolescent, Multicenter cross-sectional study, Prevalence, Impact factors

## Abstract

**Background:**

The aim of this study was to assess the environmental impact-factors differences between female and male HT in the eastern Chinese adolescents.

**Methods:**

We used a multicenter, cross-sectional study to estimate the prevalence rates and the associated impact-factors of hysteria tendency (HT) in 2014. Totally, 10131 adolescents took part in the study from three School Health Surveillance System centers in three provinces of east China. The data were collected using a common protocol and questionnaire in order to identify common environment affecting in this population.

**Results:**

An overall positive rate of HT among the eastern Chinese adolescents was 13.13% (95% CI: 12.48%–13.80%) with 14.01% (95% CI: 13.05%–15.02%) for females and 12.30% (95% CI: 11.43%–13.22%) for males. Gender-stratified regression analyses revealed that 7 out of the 21 tested covariates were linked to HT only in males, while 9 out of the 21 tested covariates were associated with female HT only. Although, the models pointed out that of all independent variables, the variable –family medical history was the strongest environment impact to both the male HT (amOR=2.49, 95% CI=1.77–3.25) and female HT (amOR=2.83, 95% CI=2.19–3.68).

**Conclusion:**

HT is prevalent among adolescents in the eastern Chinese adolescents. Environmental factors differences between female and male HT are significant in adolescents, and HT affects more female than male. First, prevention and therapy of HT in adolescents should focus on various social, school and family environment settings, and individual characteristics. Second, gender -respective intervention programs against HT in adolescents should be implemented.

## Introduction

The history of hysteria that goes back to ancient times (1). It had ever been referred to as “conversion disorder”, “psychogenic disorder”, “non-organic disease”, “functional disease”, or “medically unexplained disease”(2). As originally described in the Diagnostic and Statistical Manual of Mental Disorders (fifth edition) (DSM-V), the hysteria, also called “somatic symptom disorders (SSD)”(3). Based on the biopsychosocial model of human behavior, researches have suggested the complex between the body, mind, and sociocultural environments for hysterics (4).

Hysteria is part of the most prevalent diseases globally, which the prevalence of hysteria is 1%– 4% in the general population and 10% or more within psychiatric settings (5–7). Like depression and anxiety, hysteria is a symptom of emotional disturbance and mental disorder that are common among children and adolescents (8). Studies argue that the hysteria may result in significant social, economic, and health burdens in children and adolescents (9). In addition, hysteria has a major impact on functioning and quality of life, and affects anyone often in early life and for sustained periods, causing numerous disease years (10).

In recent decades, there has been a growing awareness of hysteria in childhood and adolescence (8). Increased recognition of the presence of hysteria has led to a growing interest in the etiology, comorbidities, and outcomes of early-onset hysteria (such as hysterical behavioral tendencies, which is traditionally interpreted as a preexisting personality trait defined by the dual characteristics of hysterical conversion, and high hysteria scores with no clinical symptoms) in young people (11,12). Historically, a considerable body of clinical literature has been working on the description and treatment of hysterical behavioral tendencies (11, 12). This hysteria tendency (HT) has been explored from various perspectives: (a) the meaning of hysterical symptomatology; (b) the interpersonal influence tactics; (c) the cognitive style; (d) and the characteristic misconceptions of the hysterical individual; to name but a few. With few exceptions, however, the focus has been on HT in abstraction of the environmental contexts in which they occur. The HT has been discussed as an isolated entity apart from environmental reasons that might well render his or her behavior characteristics more intelligible, as well as more manageable and preventable. Moreover, some literatures show that the impact of stress and its related disorders and socioeconomic effects is felt by the individuals (health and socioeconomic), the families, schools and even the individuals’ personality characteristics (13,14). Meanwhile, related researches also prove that HT was a strong risk for depression, anxiety, common musculoskeletal disorders, acting independently of other aspects of personal mental health(15,16). Hence, this would have serious implications for their investigation and prevention.

Prevalence rates of the HT vary widely depending on the studied population and the set of symptoms being employed to diagnosis. So far, no specific prevalence estimates for adolescents have been available. In addition, although female gender, rural settings, lower education level, lower socioeconomic status, marital status, having a family member with the disease, and childhood sexual abuse are relevant to an increased risk for HT (14–16), a broad range of potential risk including various potential modifiable ones associated with this condition should be investigated in large-scale epidemiological studies to develop effective interventions against this disorder.

The purpose of the present study was to supplement this large body of clinical literature that has viewed primary characteristics of hysteria from the population perspective. This will be done in a threefold manner. First, we designed this study to focus on environmental effects on gender-specific characteristics of the adolescents’ HT. Second, we conducted a multicenter cross-sectional study to report the prevalence rates of HT in the eastern Chinese adolescents. Third, and finally, environmental impact-factors differences between female and male HT will be identified by unconditional multiple regression analyses in this population.

## Materials and Methods

### Study design

**Fig. 1:**
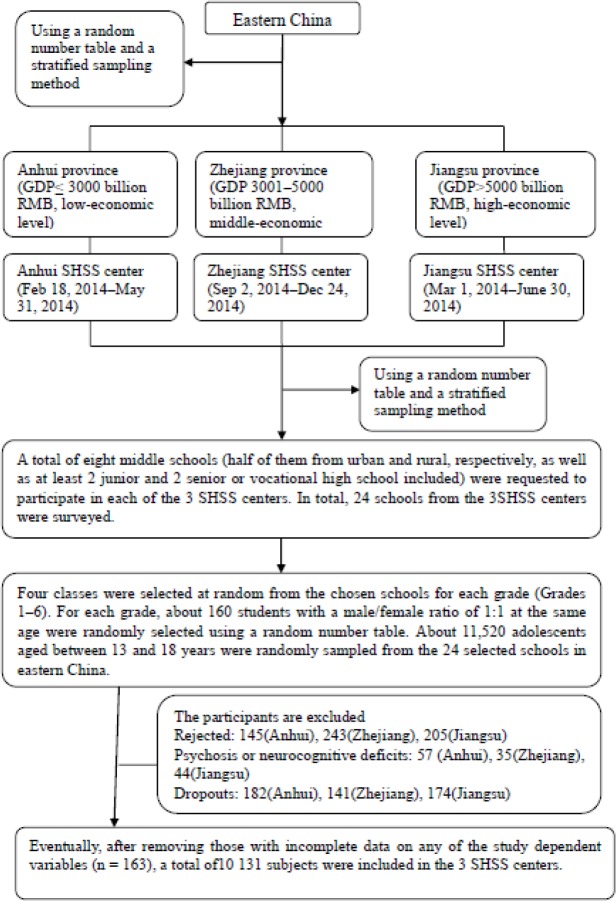
The flow chart of data sampling. *GDP* gross domestic product, *SHSS* school health surveillance system, *HT* hysterical tendencies. The prevalence of adolescents’ HT seems to be disconnected with the economic level in the present study (*P*> 0.05)

Our research design was a school-based cross-sectional multicenter study in three School Health Surveillance System (SHSS) centers from three provinces (Anhui, Jiangsu and Zhejiang) of China. In 2014, a multiphase stratified cluster sampling approach produced a representative sample of 13- to 18-year-old students who were attending public middle schools (including junior, senior, and vocational high schools) in the studied regions (Fig. 1). Trained field-workers managed two questionnaires to the subjects assessed as having HT and environment influence of HT. Face-to-face interviews were undertaken in each of the 3 SHSS centers by interviewers trained by the field-workers.

### Measures and procedure The adolescents’ HT measurement

This study designed a two-stage appraisal procedure to identify the adolescents’ HT. In the first stage, the HT was screened by the MMPI-2 Hy (Hysteria) Scale (17). Internal consistency in the present sample was 0.89 (Cronbach coefficient). The inventory was scored by the computer software. The raw scores of the scale are converted to a uniform T-score according to the transformation rule (18). Furthermore, researchers summed up the responses. In the second stage, the individuals who responses to the questionnaire suggested they might have HT were further examined by three psychologists for a final assessment. Among which, the assessment of HT were based on the DSM- fourth edition and Chinese classification of mental disorders. Besides, three psychologists carefully reviewed the diagnostic criteria of hysteria and related studies of HT and estimated suspected adolescents’ HT respectively. To ensure consistency of assessment of different psychologists, a mutual evaluation form was used, and only when HT was consistently diagnosed by all three psychologists could be adolescents’ HT be confirmed. A case was defined as ‘HT’ if a subject: (a) had an uniform T-score ≥ 60 (19), and in those with hysterical behavioral tendencies (i.e., a) an egoistic person, involuntary behavior, strong beliefs and extensive presentation of self, nor responsibility for, one’s own behavior; b) immature, histrionic, provocative, and attention-seeking modes of self-presentation; c) labile and extreme, but superficial, emotionality; and d) suggestible, dependent demandingness in situations); (b) had no history of psychosis or neurocognitive deficits; (c) only cases with intact intellectual functioning (intelligence quotient >70).

### Environmental characteristics measurement

We designed one questionnaire to assess environmental causes contributing to HT. The main contents of questionnaire survey include four aspects, social demographics (e.g., gender, age, ethnicity, address), individual characteristics (e.g., learning achievement, attitude and motivation, left-behind adolescents, somatotype [regarding an overall outlook of the body and conveys a meaning of morphological features of the human body, such as ineligible includes obesity, overweight and marasmus and so on]), family factors (e.g., family income and size, parents’ education and occupation [including stable (such as peasants, workers, teachers, doctors or cadres, other formal professions), unstable (referring to temporary worker, self-employed), no (comprising of no formal professions and unemployed)], family medical history [history of psychosis or neurocognitive diseases in your family]), and school factors (e.g., boarding or not, administration model, class size).

The reliability of the questionnaire was tested with 56 individuals with HT in Hangzhou City, Zhejiang Province, which was not included in this research project. We used Cronbach’s alpha coefficient to assess the responses. The results revealed that internal consistency was 0.83 for socio-demographic characteristics, 0.87 for family environmental factors, 0.84 for school environmental factors, and 0.88 for individual personality characteristics, respectively. Our findings suggested the validity of the designed questionnaire can meet the needs of the study.

### Assessment of anxiety and depression

The present study used the Hospital Anxiety and Depression Scale (HADS) to evaluate anxiety and depression (20). The HADS has been specifically developed for detection of anxiety and depression in patients with somatic conditions. It is structured in an anxiety subscale (HADS-A) and a depression subscale (HADS-D) both containing seven items, rated 0–3, giving a possible maximum score for anxiety and depression of 21. Scores < 8 mark no clinical distress; scores from 8 to 10 indicate possible psychiatric morbidity; and scores ≥11 show probable pathological levels of distress (20). Prior studies have demonstrated the internal consistency and reliability of the Chinese versions of the HADS (21, 22). Internal consistency in the present sample was 0.86.

### Ethical statement

The study protocol was subject to approval by the Ethics Committees of School of Public Health, Medical College of Soochow University, and by the Ethics Review Boards in each of the participating cities. Details of the study were explained to all participants or guardians who provided a written informed consent prior to be involved in the study and completed the experiment individually in each SHSS center.

### Statistical analysis

All data were double-entered, verified, and de-identified in the Epidata open-source database (version 3.1). We analyzed the data using R version 3.2.2. Imputation of missing data was done separately for each center and three imputed datasets were created.

We used descriptive statistics to calculate the frequency and percentages for categorical variables, and mean (M) ± standard deviation (SD) for normally distributed continuous variables.

We used univariate analyses to separately select all interested covariates that were potentially associated with HT for entering the subsequent multiple regression models at a significance level (alpha) of 0.05. Briefly, independent t tests were used for the continuous variables with a normal distribution; Bartlett’s one-way ANOVA for three or more variables with both a normal distribution and homogeneity of variance; Pearson Chi-square test categorical independent variables, and univariate Kruskal-Wallis nonparametric tests for three or more unpaired categorical variables.

We conducted unconditional multiple logistic regression model to evaluate whether all covariates including social demographics, family and school environmental factors, as well as individual personality that had been selected by the univariate analyses were associated with HT. A stepwise procedure was used to further select the covariates that were associated with HT at a significance level of *P*>0.10 for removal and *P*<0.05 for reentry. The final model was selected according to the minimum statistics of the Akaike information criterion. Hypothesis testing was conducted using a two-sided test, with an alpha value of 0.05 to indicate statistical significance.

## Results

### Baseline characteristics of all subjects and the positive rates of HT in the adolescents

A response rate of 89.36% was obtained and 10294 questionnaires completed twenty-four selected schools. After removing those with incomplete data on any of the study dependent variables (n = 163, accounting for 1.58%), 10,131 participants were retained in the subsequent analyses. Kappa scores for test–retest reliability of the survey ranged from 0.89 to 0.92.

Table 1 summarized the principal locations and dispersions of the uniform T-scores among different baseline characteristics of all subjects. Statistically differences of the uniform T-score and positive rates were found for the variables of gender and age (*P* <0.05), but not for the variables of nationality (Han or Ethnic Minorities), residence area (rural or urban), and sampling area (*P*>0.05) (Table 1).

**Table 1:** Central locations and dispersions of the uniform T-scores and positive rates of HT among different baseline characteristics of all subjects (N=10131)

***Variables***	***Total (N=10131)***	***Uniform T-score (M±SD)***	***P value***	***HT (n=1330)***	***Positive Rates of HT % (95% CI)***	***P value***	***OR(95% CI)***
Gender							
Female	4881(48.18)	50.93±9.48	**<0.001^a^**	684	14.01(13.05–15.02)	**0.011^c^**	**1.16(1.03–1.31)**
Male	5250(51.82)	49.03±10.43		646	12.30(11.43–13.22)		
Age (yr)							
13	2308(22.78)	49.66±10.17	**0.003^b^**	286	12.39(11.07–13.81)	**0.036^d^**	NA
14	2251(22.22)	49.78±10.00		270	11.99(10.68–13.41)		
15	1663(16.41)	51.39±10.19		241	14.49(12.83–16.28)		
16	1631(16.10)	49.80±10.11		190	11.65(10.13–13.31)		
17	1120(11.06)	49.81±9.79		158	14.11(12.12–16.28)		
18	1158(11.43)	50.44±8.96		185	15.98(13.91–18.22)		
Nationality							
Han	10023(98.93)	49.98±10.00	0.451^a^	1316	13.13(12.47–13.81)	0.959^d^	1.01(0.57–1.93)
Ethnic minorities	108(1.07)	50.71±10.01		14	12.96(7.27–20.79)		
Residence area							
Rural	5353(52.84)	50.09±10.09	0.394^a^	731	13.66(12.75–14.60)	0.068^c^	1.11(0.99–1.25)
Urban	4778(47.16)	49.92±9.92		599	12.54(11.61–13.61)		
Sampling area(province)							
Anhui	3643(35.96)	49.69±9.52	0.242^b^	452	12.41(11.35–13.52)	0.181^d^	NA
Zhejiang	3025(29.86)	50.05±10.22		397	13.12(11.94–14.38)		
Jiangsu	3463(34.18)	49.89±9.65		481	13.89(12.75–15.09)		

Data expressed as n(%);uniform T-score according to formula conversion methods for standardization /Bold values are those that reach statistical significance (*P*<0.05) /HT; hysteria tendency, M±SD; mean ± standard deviation, OR; odds ratio, CI; confidence interval

a Independent t tests

b Bartlett’s one-way ANOVA

c Pearson Chi-square test

d Kruskal-Wallis nonparametric tests

As showed in Table 1, frequency distributions and positive rates of HT were presented among different baseline characteristics of all subjects. An overall positive rate of HT among the eastern Chinese adolescents was 13.13% (95% CI=12.48%–13.80%) with 14.01% (95% CI =13.05%–15.02%) for females and 12.30% (95% CI=11.43%–13.22%) for males.

### Impact-factors associated with HT in the adolescents

The univariate analyses showed about 21 out of the 35 tested covariates were associated with HT, separately (*P* <0.01), and these significant covariates were: 1) the socio-demographic such as gender and age, 2) the family environmental factors including family income, father’s occupation, parents’ marital status, rearing style, and family medical history, 3) the school environmental factors comprising boarding school, new student, school administration model, compliance with the school regulations, and school mental health education, and 4) the individual characteristics like learning attitude and motivation, somatotype, attitudes towards criticism, superstitious beliefs, left-behind adolescents, learning achievement, HADS-A score and HADS-D score. The remaining 14 covariates including residence area, nationality, sampling area, education level of parents, mother’s occupation, number of family members, class size, class staff, parents’ religion faith, trouble with studying, attitudes towards being bullied, self-care situation, and only child did not significantly relate to the HT risk. Ultimately, only those significant covariates were considered in the next multiple regression modeling.

Table 2 provides a summary of a final multiple regression models to regress HT on the manifold impact-factors with a stepwise procedure for further variable selections. In the final model, about 10 covariates including gender, parents’ marital status, rearing style, family medical history, school administration model, school mental health education, somatotype, left-behind adolescents, HADS-A score and HADS-D score were associated with the HT (Table 2). The model noted that of all independent variables, the variable – family medical history was the strongest risk factor to the HT (OR=2.64, 95% CI =2.21–3.16) (Table 2). To further explore the possible gender difference in the association between HT and risk factors. We performed gender-stratified multiple regression analyses.

**Table 2:** Analyses of unconditional multiple logistic regression of HT on HT-associated risk factors

***Variables***	***Overall***		***Female***		***Male***	
***Socio-demographic characteristics***	***OR(95%CI)***	***P value***	***OR(95%CI)***	***P value***	***OR(95%CI)***	***P value***
Gender	**1.43(1.24–1.75)**	**<0.001**	NA	NA	NA	NA
Age	1.12(0.98–1.87)	0.082	**1.17(1.03–1.94)**	**0.019**	1.08(0.91–1.74)	0.358
Family environmental factors						
Father’s occupation	0.90(0.87–1.41)	0.194	0.90(0.75–1.03)	0.089	1.00(0.90–1.12)	0.954
Family income	1.13(0.91–1.42)	0.367	1.09(0.93–1.64)	0.548	1.24(0.98–1.62)	0.075
Parents’ marital status	**1.20(1.02–1.64)**	**0.006**	**1.29(1.05–1.69)**	**0.002**	1.12(0.99–1.13)	0.138
Rearing style	**1.27(1.03–1.55)**	**0.022**	**1.51(1.16–1.98)**	**0.003**	1.03(0.75–1.40)	0.864
Family medical history	**2.64(2.21–3.16)**	**<0.001**	**2.83(2.19–3.68)**	**<0.001**	**2.49(1.77–3.25)**	**<0.001**
School environmental factors						
Boarding school	1.37(0.94–1.65)	0.111	1.19(0.91–1.54)	0.199	1.56(0.91–1.85)	0.081
New student	1.09(0.91–1.38)	0.441	1.17(0.94–1.42)	0.185	1.04(0.96–1.09)	0.613
School administration model	**1.39(1.11–1.97)**	**<0.001**	1.11(0.94–1.33)	0.195	**1.54(1.13–1.84)**	**<0.001**
Compliance with school regulations	0.89(0.71–1.29)	0.335	0.87(0.74–1.02)	0.085	0.95(0.91–1.06)	0.554
School mental health education	**0.69(0.61–0.89)**	**<0.001**	**0.77(0.64–0.91)**	**0.015**	**0.64(0.53–0.88)**	**<0.001**
Individual characteristics						
Learning attitude	1.05(0.92–1.59)	0.119	1.15(0.97–1.34)	0.088	0.99(0.94–1.39)	0.201
Learning motivation	1.18(0.95–1.43)	0.193	1.08(0.93–1.33)	0.094	1.26(0.97–1.52)	0.222
Somatotype	**1.42(1.15–1.74)**	**0.011**	**1.60(1.12–1.78)**	**0.004**	1.11(0.91–1.36)	0.307
Attitudes towards criticism	1.18(0.94–1.43)	0.097	1.06(0.88–1.28)	0.055	1.28(0.97–1.62)	0.129
Left-behind adolescents	**1.63(1.12–1.88)**	**0.001**	**1.66(1.23–1.96)**	**<0.001**	**1.57(1.03–1.81)**	**0.002**
Superstitious beliefs	1.16(0.90–1.53)	0.097	1.04(0.91–1.35)	0.129	**1.29(1.07–1.66)**	**0.035**
Learning achievement	1.16(0.84–1.56)	0.231	1.10(0.93–1.64)	0.093	1.23(0.73–1.71)	0.349
HADS-A score	**2.01(1.14–3.11)**	**0.004**	**2.23(1.82–3.86)**	**<0.001**	**1.89(1.27–2.81)**	**0.002**
HADS-D score	**1.98(1.44–2.93)**	**0.002**	**1.92(1.58–3.15)**	**0.005**	**2.11(1.60–3.02)**	**<0.001**

HT; hysteria tendency, OR; odds ratios, CI; confidence intervals, NA; not available

The results revealed that age, parents’ marital status, rearing style, family medical history, school mental health education, somatotype, left-behind adolescents, HADS-A score and HADS-D score were significantly linked to HT only in females, while family medical history, school administration model, school mental health education, left-behind adolescents, superstitious beliefs, HADSA score and HADS-D score were significantly associated with male HT only; and five variables including family medical history, school mental health education, left-behind adolescents, HADSA score and HADS-D score were related to HT in both (Table 2).

The models also suggested that of all independent variables, the variable – family medical history was the strongest environment impact to both the male HT (OR=2.49, 95% CI=1.77–3.25) and female HT (OR=2.83, 95% CI= 2.19–3.68) (Table 2).

## Discussion

To our best knowledge, this study is the first to investigate the prevalence rate of HT among a large sample of Chinese adolescents. Our findings prove that HT is prevalent among adolescents in East China. Adolescent HT may become a serious public health problem in East China. Previous studies have shown that some Asians have a high tendency to express their psychological or emotional problems in somatic terms, not only in psychiatric illnesses, but also in an ordinary conversation (23, 24). The current finding shows the prevalence of HT was 13.13% in the eastern Chinese adolescents. The overall prevalence of HT was high, and similar to the prevalence of 14% reported in Saudi Arabians (25). Our finding contrasts, however, with two other studies of HT (26) reported HT (somatoform symptoms) in only 0.3% of their sample in Germany, and reported HT (unexplained symptoms) in only1.3% of their sample in the United States (27). The analysis of geographical distribution showed some differences, thus emphasizing the role of socio-cultural and socio-economic variables in co-determining the onset and course of HT. Our data on frequencies of HT in the economic level are in line with some of the results of mass hysteria studies (28), showing HT is more frequent in underdeveloped areas.

Our result found that females were more likely than males to suffer from HT, which is consistent with another study (29). This might be because the psychological maturity of female adolescents was more vulnerable than in male adolescents (30). The present research suggested that boys perceive greater school support than girls, while family support from parents is an important determinant on which depends on whether and how many girls are dealing with mental problems.

Some modifiable HT-associated impact factors such as parents’ marital status, rearing style, the school administration model, school mental health education, somatotype, left-behind adolescents, HADS-A score and HADS-D score have been identified in our study. Correlation analysis showed there is a relationship between marital satisfaction and children’s psychological compatibility and child adjustment style and character characteristics of parents play the variable modifying role (31). Research also revealed that most of the children with somatic complaints and somatization disorders are from inconstant families, and underwent misbehaviors (32). In adolescents, the influences of anxiety and depression on mental health have been proven already (33). These research findings are in mutual accord with our study results. Based on our findings, it would be of particular benefit to positively reduce HT prevalence in adolescents by modifying some impact-factors in environmental settings and individual characteristics to prevent hysteria occurring.

In our study, significant associations with female HT were found in age, parents’ marital status, rearing style, and somatotype. This finding is obviously different from male HT. One possible reason was that boys and girls showed certain differences with biological and environmental factors implicated in the development of mental problems (34). Another might be that in comparison with male adolescents, females depended more on the parents’ support. Age difference of adolescents’ HT was found in both gender, one possibility is that age is a crucial factor, and higher rates of psychological symptoms in females are detected at mid-puberty through adult life, as opposed to a male preponderance until early adolescence (35). There was significant relationship between parental rearing style (authoritarian, democratic, and permissive style) and mental health as if females were more frequently affected by their mother’s parenting skills (36). In a study on somatotype and mental health, Mantarkov *et al.* described there are significant gender-specific differences in the somatotype of bipolar disorder patients as though females of ineligible somatotype could be more easily violated by psychological problems (37). These findings provide evidence for improving hysteria prevention strategy by targeting supervision on high risk factors of HT in different gender adolescents.

Studies confirm that family medical history, school mental health education, left-behind adolescents, HADS-A score and HADS-D score were associated with both male and female HT. A study suggested the unique platform that schools can offer in access to and support for children and adolescents with psychological difficulties, has led to an expansion of school-based mental health interventions (38). In addition, most research that has found out school mental health resources, particularly those related to early identification, may influence sector of service use for youths with mental disorders (39). The results of our research showed the parents working outside the home can influence on psychological behavior among adolescents from both genders. The parents’ role in promoting mental health among their children can play a key role, such as the transfer of positive attitudes and values, and sparing their pain and pressure through frequent exchange and discussion (40). The present study found that anxiety score, depression score was significantly related to the prevalence of HT as if they can increase HT severity in adolescents, like another study (41). One explanation could be that anxiety and depression with possible genetic and environmental risk factors contributed to SSD in children and adolescents (42). From these results, the benefits of mental health promotion and other protection strategies (such as family support programs or medical assistance programs) could be beneficial for adolescents’ HT.

Most importantly, this study suggests that family medical history was the strongest risk factor in the HT in both males and females. One reason this may be the case is that HT is a complex disease lead by genetic and environmental factors. Several lines of evidence suggested that family medical history has historically served as an important validator for definitions of psychiatric disorders (43). In this discussion, we focus on family medical history as a significant impact factor for HT in adolescents because the associations are stronger for HT than controls. This finding is consistent with reports from other studies linking family history with offspring mental disorder (44). Data shows that family medical history not only increases the risk of mental disorder among high-risk populations but also increases the relapse of mental illness (45). As such, early identification and intervention of family history in adolescents could prevent the occurrence of various kinds of adverse events, and the most significant risk factor is alarming and more specific prevention strategy is needed.

### Limitations

There are some limitations to this study. First, the study was based on a cross-sectional sample, which marks the casual relations between the HT and the risk factors cannot be determined. Second, some data in our study were self-reported and thus the underreport possibility due to social desirability cannot be excluded. However, the confidential nature of the survey in our study may have significantly decreased the under-reports. Third, because of no standard approach for the measurement of HT, the misclassification of the outcome may underestimate the associations between HT and risk factors although we may have reduced it by integrating the subjective and objective methods for the HT measurement. Fourth, only adolescents attending school were sampled and the out-of-school adolescents were not included in the study. Thus, generalizing the results from the study population to the entire adolescent population in China should be cautious.

## Conclusion

HT is prevalent (13.13%) among adolescents in East China. This multicenter cross-sectional study, for the first time in the eastern Chinese adolescents, confirmed roles of major impact factors for HT such as family medical history, HADS-A score, and HADS-D score, etc. These findings have important HT prevention and control implications for adolescents. In addition, girls unlike boys show significantly higher levels of HT as though gender differences in impact-factors of HT in adolescents are significant. As an effective measure of preventive individual and mass hysterics, early assessment and intervention for the sex-specific HT will be able to play an important role in increasing and improving the health-related quality of life (HRQoL) in adolescents.

The research results suggest the importance of preparing a national plan and program to promote mental health in order to help young people change unhealthy psychological problems and increase mental health intervention, and thus improve their health.

## Ethical considerations

Ethical issues (Including plagiarism, informed consent, misconduct, data fabrication and/or falsification, double publication and/or submission, redundancy, etc.) have been completely observed by the authors.

## References

[1] Tasca C Rapetti M Carta MG Fadda B (2012). Women and hysteria in the history of mental health. Clin Pract Epidemiol Ment Health, 8: 110–119. 23115576 10.2174/1745017901208010110 PMC3480686

[2] Feinstein A (2011). Conversion disorder: advances in our understanding. CMAJ, 183 (8):915–920. 21502352 10.1503/cmaj.110490 PMC3091899

[3] Black DW Grant JE (2014). DSM-5® guidebook: the essential companion to the diagnostic and statistical manual of mental disorders. Washington: American Psychiatric Publishing.

[4] Cavanna AE (2015). The past, present and future of hysteria. Cogn Neuropsychiatry, 20(4): 372–376.

[5] Creed FH Davies I Jackson J (2012). The epidemiology of multiple somatic symptoms. J Psychosom Res, 72(4): 311–317. 22405227 10.1016/j.jpsychores.2012.01.009

[6] Fink P Schröder A (2010). One single diagnosis, bodily distress syndrome, succeeded to capture 10 diagnostic categories of functional somatic syndromes and somatoform disorders. J Psychosom Res, 68(5): 415–426. 20403500 10.1016/j.jpsychores.2010.02.004

[7] Escobar JI Cook B Chen CN (2010). Whether medically unexplained or not, three or more concurrent somatic symptoms predict psychopathology and service use in community populations. J Psychosom Res, 69(1): 1–8. 20630257 10.1016/j.jpsychores.2010.01.001 PMC2905311

[8] Kozlowska K Palmer DM Brown KJ (2015). Conversion disorder in children and adolescents: a disorder of cognitive control. J Neuropsychol, 9(1): 87–108. 24405496 10.1111/jnp.12037

[9] Fritz GK Fritsch S Hagino O (1997). Somato-form disorders in children and adolescents: a review of the past 10 years. J Am Acad Child Adolesc Psychiatry, 36(10): 1329–1338. 9334545 10.1097/00004583-199710000-00014

[10] Hofstra MB Van Der Ende J Verhulst FC (2002). Child and adolescent problems predict DSM-IV disorders in adulthood: A 14-year follow-up of a Dutch epidemiological sample. J Am Acad Child Adolesc Psychiatry, 41(2): 182–189. 11837408 10.1097/00004583-200202000-00012

[11] Chodoff P Lyons H (1958). Hysteria, the hysterical personality and “hysterical” conversion. Am J Psychiatry, 114(8): 734–740. 13498192 10.1176/ajp.114.8.734

[12] Halleck SL (1967). Hysterical personality traits: Psychological, social, and iatrogenic determinants. Arch Gen Psychiatry, 16(6): 750–757. 6027372 10.1001/archpsyc.1967.01730240106015

[13] Hansen TB Steenberg LM Palic S Elklit A (2012). A review of psychological factors related to bullying victimization in schools. Aggress Violent Behav, 17(4): 383–387.

[14] Obimakinde AM Ladipo MM Irabor AE (2015). Familial and socio-economic correlates of somatisation disorder. Afr J Prim Health Care Fam Med, 7(1):10.4102/phcfm.v7i1.746. 10.4102/phcfm.v7i1.746 PMC4564913 26245602

[15] Solidaki E Chatzi L Bitsios P (2010). Work related and psychological determinants of multi-site musculoskeletal pain. Scand J Work Environ Health, 36(1): 54–61. 20011982 10.5271/sjweh.2884 PMC3242043

[16] Neeleman J Ormel J Bijl RV (2001). The distribution of psychiatric and somatic ill health: Associations with personality and socioeconomic status. Psychosom Med, 63(2):239–247. 11292271 10.1097/00006842-200103000-00007

[17] Zhang J Song W Cheung FM (2004). The Chinese Minnesota Multiphasic Personality Inventory-2 (MMPI-2) (Chinese language edition). Beijing: Geological Press. (In Chinese).

[18] Tellegen A Ben-Porath Y (1992). The new uniform t-scores for the MMPI-2: Rationale, derivation, and appraisal. Psychol Assess, 4(2): 145–155.

[19] Yung YF Chan W Cheung FM (2000). Standardization of the Chinese Personality Assessment Inventory: The prototype standardization method and its rationale. Asian J Soc Psychol, 3(2): 133–152.

[20] Bjelland I Dahl AA Haug TT Neckelmann D (2002). The validity of the Hospital Anxiety and Depression Scale: an updated literature review. J Psychosom Res, 52(2):69–77. 11832252 10.1016/s0022-3999(01)00296-3

[21] Leung CM Wing YK Kwong PK (1999). Validation of the Chinese-Cantonese version of the Hospital Anxiety and Depression Scale and comparison with the Hamilton Rating Scale of Depression. Acta Psychiatr Scand, 100(6):456–461. 10626925 10.1111/j.1600-0447.1999.tb10897.x

[22] Hong JS Tian J (2014). Prevalence of anxiety and depression and their risk factors in Chinese cancer patients. Support Care Cancer, 22(2): 453–459. 24091720 10.1007/s00520-013-1997-y

[23] Zhang AY Snowden LR Sue S (1998). Differences between Asian and White Americans’ help seeking and utilization patterns in the Los Angeles area. J Community Psychol, 26(4): 317–326.

[24] Kawanishi Y (1992). Somatization of Asians: An artifact of Western medicalization? Transcultural Psychiatric Research Review, 29(1): 5–36.

[25] Alqahtani MM Salmon P (2008). Prevalence of somatization and minor psychiatric morbidity in primary healthcare in Saudi Arabia: a preliminary study in Asir region. J Family Community Med, 15(1): 27–33. 23012164 PMC3377053

[26] Rief W Hessel A Braehler E (2001). Somatization symptoms and hypochondriacal features in the general population. Psychosom Med, 63(4):595–602. 11485113 10.1097/00006842-200107000-00012

[27] Swartz M Blazer D Woodbury M (1986). Somatization disorder in a US southern community: use of a new procedure for analysis of medical classification. Psychol Med, 16 (3): 595–609. 3763774 10.1017/s0033291700010357

[28] Colligan MJ Pennebaker JW Murphy LR (2013). Mass psychogenic illness: A social psychological analysis. London: Routledge.

[29] World Health Organization (WHO) (2016). Risks to mental health: an overview of vulnerabilities and risk factors.

[30] Lewis AJ Kremer P Douglas K (2015). Gender differences in adolescent depression: Differential female susceptibility to stressors affecting family functioning. Aust J Psychol, 67(3): 131–139.

[31] Brock RL Kochanska G (2015). Decline in the quality of family relationships predicts escalation in children’s internalizing symptoms from middle to late childhood. J Abnorm Child Psychol, 43(7): 1295–1308. 25790794 10.1007/s10802-015-0008-9 PMC4561594

[32] Bolghan-Abadi M Kimiaee SA Amir F (2011). The relationship between parents’ child rearing styles and their children’s quality of life and mental health. Psychology, 2(3): 230–234.

[33] Coles ME Ravid A Gibb B George-Denn D (2016). Adolescent mental health literacy: young people’s knowledge of depression and social anxiety disorder. J Adolesc Health, 58(1): 57–62. 26707229 10.1016/j.jadohealth.2015.09.017

[34] Fattore L Melis M Fadda P Fratta W (2014). Sex differences in addictive disorders. Front Neuroendocrinol, 35(3): 272–284. 24769267 10.1016/j.yfrne.2014.04.003

[35] Piccinelli M Wilkinson G (2000). Gender differences in depression. Br J Psychiatry, 177: 486–492. 11102321 10.1192/bjp.177.6.486

[36] Patock-Peckham JA Cheong JW Balhorn ME Nagoshi CT (2001). A social learning perspective: a model of parenting styles, self-regulation, perceived drinking control, and alcohol Use and problems. Alcohol Clin Exp Res, 25(9): 1284–1292. 11584147

[37] Mantarkov M Ahmed-Popova F Akabaliev V Sivkov S (2016). Somatotype in Bipolar Disorder Revisited: Gender Differences, Neuro-development and Clinical Implications. Imp J Interdiscip Res, 2(9):1028–1037.

[38] Fazel M Hoagwood K Stephan S Ford T (2014). Mental health interventions in schools in high-income countries. Lancet Psychiatry, 1(5): 377–387. 26114092 10.1016/S2215-0366(14)70312-8 PMC4477835

[39] Green JG McLaughlin KA Alegría M (2013). School mental health resources and adolescent mental health service use. J Am Acad Child Adolesc Psychiatry, 52(5): 501–510. 23622851 10.1016/j.jaac.2013.03.002 PMC3902042

[40] Huang Y Zhong XN Li QY (2015). Health-related quality of life of the rural-China left-behind children or adolescents and influential factors: a cross-sectional study. Health Qual Life Outcomes, 13: 29. 25888732 10.1186/s12955-015-0220-x PMC4349722

[41] Campo JV (2012). Annual Research Review: Functional somatic symptoms and associated anxiety and depression–developmental psychopathology in pediatric practice. J Child Psychol Psychiatry, 53(5): 575–592. 22404290 10.1111/j.1469-7610.2012.02535.x

[42] Nugent NR Tyrka AR Carpenter LL Price LH (2011). Gene–environment interactions: early life stress and risk for depressive and anxiety disorders. Psychopharmacology (Berl), 214(1): 175–196. 21225419 10.1007/s00213-010-2151-x PMC3615637

[43] Sullivan PF Magnusson C Reichenberg A (2012). Family history of schizophrenia and bipolar disorder as risk factors for autism. Arch Gen Psychiatry, 69(11): 1099–1103. 22752149 10.1001/archgenpsychiatry.2012.730 PMC4187103

[44] Repetti RL Taylor SE Seeman TE (2002). Risky families: family social environments and the mental and physical health of offspring. Psychol Bull, 128(2): 330–366. 11931522

[45] Rasic D Hajek T Alda M Uher R (2014). Risk of mental illness in offspring of parents with schizophrenia, bipolar disorder, and major depressive disorder: a meta-analysis of family high-risk studies. Schizophr Bull, 40 (1): 28–38. 23960245 10.1093/schbul/sbt114 PMC3885302

